# Assessing the Occurrence of Hypertension in Patients Receiving Calcitonin Gene-Related Peptide Monoclonal Antibodies for Episodic and Chronic Migraine: A Systematic Review

**DOI:** 10.7759/cureus.90244

**Published:** 2025-08-16

**Authors:** Shradha P Kakde, Khurram Islam, Muhammad Faizan Ali, Muhammad Shakaib Anwar, Laiba Nadeem, Abdul Rauf Rana, Abdul Wahab Rana, Sardar Khizar Hayat, Syed Momin Ali, Nikhil Deep Kolanu

**Affiliations:** 1 Medicine and Surgery, Mahatma Gandhi Medical College and Research Institute, Aurangabad, IND; 2 Internal Medicine, Allied Hospital Faisalabad, Faisalabad, PAK; 3 Internal Medicine, Central Park Medical College, Lahore, PAK; 4 Ophthalmology, Rawal Institute of Health Sciences, Islamabad, PAK; 5 Medicine and Surgery, King Edward Medical University, Lahore, PAK; 6 Internal Medicine, Ameer-ud-Din Medical College, Lahore, PAK; 7 Internal Medicine, Milton Keynes University Hospital, Milton Keynes, GBR; 8 Internal Medicine, King Edward Medical University, Lahore, PAK; 9 Internal Medicine, China Medical University, Shenyang, CHN

**Keywords:** adverse events, blood pressure, cardiovascular safety, cgrp monoclonal antibodies, erenumab, fremanezumab, galcanezumab, hypertension, migraine prevention, systematic review

## Abstract

Calcitonin gene-related peptide (CGRP) monoclonal antibodies have emerged as effective preventive treatments for episodic and chronic migraine, but concerns have been raised regarding their potential cardiovascular safety profile, particularly the risk of hypertension. This systematic review aimed to assess the occurrence of hypertension in patients receiving CGRP monoclonal antibodies for migraine prevention. A comprehensive search of PubMed, Embase, Scopus, and Cochrane Library was conducted for studies published between January 2015 and June 2025. Five studies met the inclusion criteria, encompassing 75,065 patients across randomized controlled trials, observational studies, and real-world cohort analyses. The evidence suggests a differential risk profile between CGRP receptor antagonists and ligand-targeting antibodies, with erenumab (receptor antagonist) demonstrating greater blood pressure increases compared to fremanezumab. In patients with baseline hypertension, anti-CGRP treatment was associated with a slight annual increase in antihypertensive medication requirements, while normotensive patients showed minimal blood pressure changes. Clinical trials reported low incidence rates of hypertension adverse events, though post-marketing surveillance identified 362 hypertension events in over 245,000 patient-years of exposure. The temporal pattern suggests cumulative rather than immediate effects, with a small proportion of normotensive patients requiring antihypertensive treatment after erenumab initiation. These findings indicate that while the overall risk of hypertension appears modest, clinically meaningful effects on blood pressure regulation may occur, particularly in patients with pre-existing hypertension. Regular cardiovascular monitoring is recommended for patients receiving CGRP monoclonal antibodies, especially those with baseline cardiovascular risk factors.

## Introduction and background

Migraine is a highly prevalent and disabling neurological disorder characterized by recurrent attacks of moderate to severe headache, often accompanied by nausea, photophobia, and phonophobia [1]. Globally, it is one of the leading causes of years lived with disability, affecting over one billion people and disproportionately impacting individuals during their most productive years [2]. Migraine is broadly categorized into episodic migraine (EM), defined as fewer than 15 headache days per month, and chronic migraine (CM), defined as 15 or more headache days per month for at least three months, with at least eight days per month fulfilling criteria for migraine [3]. Effective long-term prophylactic therapies for both EM and CM have remained elusive for many patients due to limited efficacy, intolerable side effects, or contraindications of traditional medications such as beta-blockers, tricyclic antidepressants, and antiepileptics.

In recent years, the pathophysiological role of calcitonin gene-related peptide (CGRP), a potent vasodilatory neuropeptide involved in the trigeminovascular system, has been extensively elucidated in migraine [4]. Elevated levels of CGRP have been observed during migraine attacks, and infusion of CGRP can trigger migraine-like headaches in susceptible individuals. These findings have catalyzed the development of a new class of preventive therapies targeting the CGRP pathway. Monoclonal antibodies (mAbs) against CGRP or its receptor, including erenumab, fremanezumab, galcanezumab, and eptinezumab, have shown promising efficacy in reducing migraine frequency and improving quality of life with favorable tolerability profiles [5].

Despite their clinical utility, concerns have emerged regarding the cardiovascular safety of CGRP-targeting therapies. CGRP is known to play a compensatory vasodilatory role in response to hypertension and ischemia by promoting vascular relaxation and increasing blood flow [6]. Inhibiting CGRP signaling may theoretically impair these protective mechanisms, potentially predisposing individuals to elevated blood pressure or hypertensive episodes. Although pivotal clinical trials for CGRP mAbs reported low incidences of hypertension, real-world pharmacovigilance data and post-marketing surveillance reports have begun to document cases of new-onset hypertension or exacerbation of pre-existing hypertension in patients receiving these agents, particularly erenumab. These reports have prompted updates in prescribing information and heightened interest in the cardiovascular safety profile of CGRP mAbs [5,7].

The potential association between CGRP monoclonal antibody therapy and hypertension carries important clinical implications, particularly for migraine patients with pre-existing cardiovascular risk factors or comorbid hypertension. However, the current evidence remains fragmented, derived from individual randomized controlled trials, observational studies, and case reports with varying definitions, reporting standards, and follow-up durations. Moreover, differences in the mechanism of action among CGRP mAbs, some targeting the CGRP ligand and others the receptor, raise the question of differential risk across agents. Given the expanding use of CGRP mAbs and the critical importance of cardiovascular safety in chronic disease management, a systematic evaluation of the available evidence is warranted. This systematic review aims to assess the occurrence of hypertension in patients receiving CGRP mAbs for episodic and chronic migraine. By synthesizing data from randomized controlled trials and observational studies, we seek to quantify the incidence of hypertension associated with these agents and evaluate potential differences across drug types, patient subgroups, and treatment durations. This evidence will inform clinical decision-making, risk-benefit assessments, and monitoring strategies in the use of CGRP-targeting therapies for migraine prevention.

## Review

Materials and methods

Study Selection

This systematic review was conducted in accordance with the Preferred Reporting Items for Systematic Reviews and Meta-Analyses (PRISMA) guidelines [8]. A comprehensive search strategy was developed to identify studies evaluating the occurrence of hypertension in patients receiving CGRP mAbs for the treatment of episodic and chronic migraine. The databases searched included PubMed, Embase, Scopus, and the Cochrane Library. Searches were restricted to studies published in English between January 1, 2015, and June 1, 2025, to capture the most up-to-date evidence since the clinical introduction of CGRP mAbs. The search strategy utilized a combination of Medical Subject Headings (MeSH) and relevant free-text keywords, such as “calcitonin gene-related peptide”, “CGRP”, “migraine”, “erenumab”, “fremanezumab”, “galcanezumab”, “eptinezumab”, “monoclonal antibody”, “hypertension”, and “blood pressure”. Reference lists of included studies and relevant reviews were manually screened to identify additional eligible articles that may not have been captured in the initial database search.

All identified records were imported into the Rayyan web application (Rayyan, Cambridge, MA), where duplicates were removed. Two independent reviewers screened the titles and abstracts to assess relevance. Studies that were considered potentially eligible based on the initial screening were retrieved in full text for detailed evaluation. Any disagreements between the reviewers regarding inclusion were resolved through discussion, and a third reviewer was consulted when necessary. A PRISMA flow diagram was used to depict the study selection process, including the number of records identified, screened, excluded, and ultimately included in the review.

Eligibility Criteria

Eligible studies included original clinical research involving human participants diagnosed with episodic or chronic migraine who received at least one CGRP mAb: erenumab, fremanezumab, galcanezumab, or eptinezumab. Studies were required to report hypertension as an outcome, either in the form of new-onset hypertension, worsening of pre-existing hypertension, elevated blood pressure readings, or hypertension-related adverse events. Eligible study designs included randomized controlled trials, prospective and retrospective cohort studies, case-control studies, and large case series. Studies were excluded if they were preclinical or animal-based, if they focused on non-migraine headache disorders without providing migraine-specific data, or if they lacked adequate reporting on hypertension outcomes. Conference abstracts without full-text data, narrative reviews, editorials, and case reports involving fewer than five patients were also excluded.

Data Extraction

Data from each included study were extracted independently by two reviewers using a standardized extraction form. Extracted variables included study characteristics (first author, publication year, country, and design), patient demographics (sample size, age, sex distribution, and migraine type), treatment details (specific CGRP mAb used, dosage, and duration of treatment), and outcomes related to hypertension. This included the incidence or prevalence of hypertension, changes in blood pressure from baseline, serious hypertension-related adverse events, and any reporting on the management or resolution of these events. Discrepancies in data extraction were resolved by consensus or through consultation with a third reviewer. If essential information was missing, attempts were made to contact the corresponding authors for clarification.

Quality Assessment

The quality of included observational studies was assessed using the Newcastle-Ottawa Scale (NOS), which evaluates studies based on three domains: selection of participants, comparability of study groups, and ascertainment of outcomes. Each study was independently scored by two reviewers, with disagreements resolved by consensus. Studies scoring ≥7 were considered high quality, 5-6 moderate, and <5 low quality.

Data Analysis

Given the variability in study design, definitions of hypertension, follow-up durations, and outcome reporting, a meta-analysis was not performed. Instead, a narrative synthesis was undertaken to summarize the findings across the included studies. The results were described qualitatively, focusing on patterns and consistency in the reported occurrence of hypertension among patients treated with CGRP mAbs. Particular attention was paid to differences in outcomes across the different types of CGRP-targeting agents, as well as any subgroup findings related to baseline cardiovascular risk or migraine subtype. The synthesis aimed to provide a coherent summary of the current state of evidence regarding this emerging safety concern.

Results

Study Selection Process

**Figure 1:**
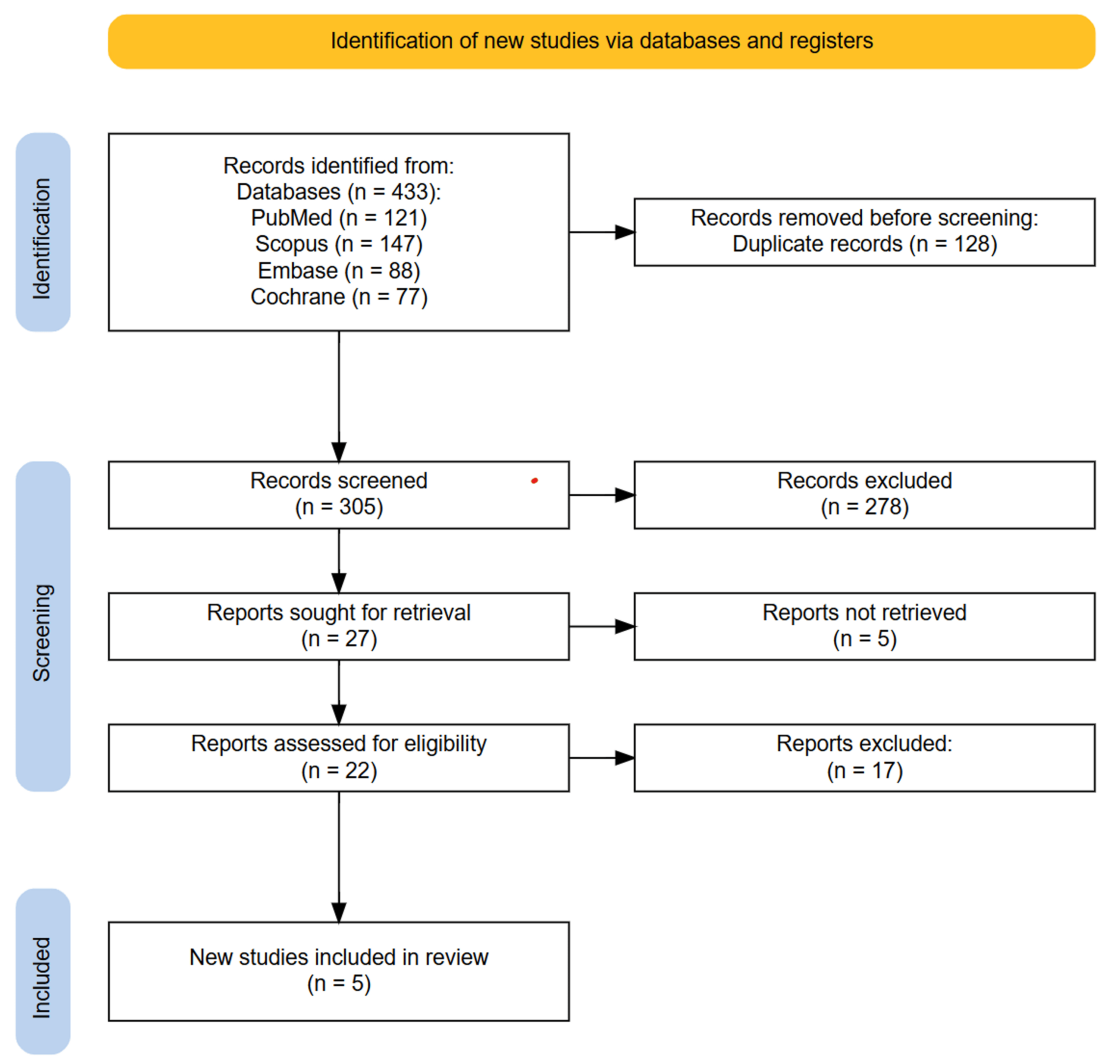
PRISMA diagram showing the study selection process. PRISMA: Preferred Reporting Items for Systematic Reviews and Meta-Analyses.

A total of 433 records were initially retrieved through database searching across PubMed, Embase, Scopus, and the Cochrane Library. Following the removal of 128 duplicates, 305 articles were screened based on their titles and abstracts by two independent reviewers using the Rayyan web application. Of these, 27 potentially relevant studies were selected for full-text assessment after meeting initial screening criteria for relevance to CGRP mAb treatment and hypertension outcomes in migraine patients. After detailed evaluation against the predetermined eligibility criteria, five studies met the inclusion requirements and were included in the final synthesis. The excluded studies primarily failed to meet criteria due to inadequate reporting of hypertension outcomes, focus on non-migraine populations, or insufficient sample sizes. No further studies were identified through manual screening of reference lists of included studies and relevant reviews. Any disagreements between reviewers during the selection process were resolved through discussion, with consultation of a third reviewer when necessary. The study selection process is illustrated in the PRISMA flow diagram (Figure 1).

Study Characteristics

The systematic review included five studies that met the eligibility criteria, encompassing diverse methodological approaches and patient populations. The studies were published between 2021 and 2024, representing the evolving evidence base for CGRP mAb safety since their clinical introduction. The included studies comprised one pooled analysis of phase 2 and 3 clinical trials (Dodick et al., 2021), one post hoc pooled analysis of four randomized controlled trials (Kudrow et al., 2023), one prospective follow-up study (de Vries Lentsch et al., 2022), one multicenter prospective real-world observational study (Mascarella et al., 2024), and one large retrospective cohort study (Wang et al., 2023) [9-13]. Sample sizes ranged considerably from 155 patients in the real-world observational study to 69,589 patients in the retrospective cohort analysis. The studies encompassed diverse age groups, with mean ages ranging from 41.3 to 41.8 years in clinical trials to 64 years in the geriatric-focused observational study. Both episodic and chronic migraine patients were represented across all studies, with chronic migraine comprising 53-69% of participants in most investigations. Wang et al.'s study specifically stratified patients by baseline hypertension status, providing insights into both hypertensive and non-hypertensive populations [13].

Three of the four FDA-approved CGRP mAbs were represented across the studies: erenumab (targeting the CGRP receptor) appeared in four studies, while fremanezumab and galcanezumab (both targeting the CGRP ligand) were examined in three studies each. Eptinezumab was not included in any of the studies. Wang et al.'s study included a broader anti-CGRP treatment category encompassing erenumab, fremanezumab, and galcanezumab, as well as the small-molecule CGRP antagonists rimegepant and atogepant. Treatment durations varied from 12-week clinical trial phases to extended real-world follow-up periods of up to five years. Standard subcutaneous dosing regimens were employed: erenumab 70-140 mg monthly, fremanezumab 225 mg monthly or 675 mg quarterly, and galcanezumab 240 mg loading dose, followed by 120 mg monthly (Table 1). The studies employed various definitions for hypertension outcomes, including blood pressure measurements, hypertension adverse events, new-onset or worsening hypertension, cardiovascular safety assessments, and initiation of antihypertensive medications. This heterogeneity in outcome definitions contributed to the decision to conduct a narrative synthesis rather than a quantitative meta-analysis.

**Table 1:** Characteristics and main findings of included studies evaluating hypertension occurrence in patients receiving CGRP monoclonal antibodies for migraine prevention. AE: adverse event; Anti-CGRP: anti-calcitonin gene-related peptide; BP: blood pressure; CGRP: calcitonin gene-related peptide; CI: confidence interval; CV: cardiovascular; ICCAE: ischemic cardiovascular and cerebrovascular adverse events; IQR: interquartile range; mAb: monoclonal antibody; mg: milligrams; NWHP: newly or worsened hypertensive patients; RR: relative risk; SD: standard deviation; T0-T4: time points 0 through 4.

Study	Year	Study type	Sample size	Age, mean, SD	Subtype of migraine (episodic or chronic)	Intervention/control	No. of patients, intervention/placebo	Quantity, dose	Route of administration	Treatment duration	Outcomes of interest assessed	Main findings	conclusions
de Vries Lentsch et al. [9]	2022	Prospective follow-up study	196	Erenumab: 42 ± 12.5 years; fremanezumab: 45 ± 12.3 years	Both episodic and chronic (53% chronic migraine overall)	Erenumab and fremanezumab	Erenumab: 109 patients; fremanezumab: 87 patients	Erenumab: 70 mg (optionally increased to 140 mg after 3 months); fremanezumab: 225 mg	Subcutaneous injection	Erenumab: every 4 weeks; fremanezumab: every 4 weeks	Blood pressure measurements at baseline and follow-up visits (T0-T4)	Systolic BP increased by 5.2 mm Hg (95% CI: 3.1-7.5), and diastolic BP increased by 3.5 mm Hg (95% CI: 2.0-4.9) at T4. Erenumab showed larger BP increases than fremanezumab. 4 patients (3.7%) with normal BP at baseline required antihypertensive treatment after erenumab.	Both systolic and diastolic BP increased after anti-CGRP (receptor) antibodies. Physicians should monitor BP in patients treated with anti-CGRP antibodies, as some may develop hypertension requiring treatment.
Dodick et al. [10]	2021	Pooled analysis of phase 2 and 3 clinical trials	2,443	41.3-41.8 ± 11.1-11.2	Both episodic and chronic migraine	Erenumab vs. placebo	Placebo: 1043; erenumab 70 mg: 893; erenumab 140 mg: 507	70 mg and 140 mg	Subcutaneous injection	12 weeks (double-blind treatment phase)	Hypertension adverse events, blood pressure measurements, and antihypertensive medication initiation	Clinical trials: Hypertension AEs similar across groups - placebo: 9/1043 (0.9%), erenumab 70 mg: 7/893 (0.8%), erenumab 140 mg: 1/507 (0.2%). postmarketing: 362 hypertension AEs in >245,000 patient-years (0.144 per 100 patient-years)	Clinical trials showed no increased risk of hypertension with erenumab vs. placebo. Postmarketing AE rates were generally low. Additional data are needed to fully characterize hypertension risk.
Kudrow et al. [11]	2023	Post hoc pooled analysis of four double-blind, randomized, multicenter trials	2,682 patients randomized across four trials; 2,443 included in pooled analysis	Adults aged 18-65 years (specific mean age not provided)	Both episodic migraine (≥4 to <15 migraine days/month) and chronic migraine (≥15 headache days/month)	Erenumab 70 mg, 140 mg vs. placebo	Short-term analysis: erenumab 70 mg (n=885), 140 mg (n=504), and placebo (n=1,032). Long-term analysis: 2,499 patients with 3,482 patient-years exposure	70 mg and 140 mg	Subcutaneous injection	12-24 weeks double-blind phase + extension phases up to 5 years	Cardiovascular safety, blood pressure changes, ischemic cardiovascular and cerebrovascular adverse events (ICCAEs)	No apparent difference in BP category worsening between placebo (14%) and erenumab groups (13% for 70 mg, 14% for 140 mg). ICCAEs were uncommon, with exposure-adjusted incidence rates of 0.4, 0.5, 0.0, and 1.1 per 100 patient-years for no risk factor, low, moderate, and high CV risk groups, respectively.	Ischemic CV and cerebrovascular adverse events were uncommon, and incidence rates were similar across 10-year CV risk categories. The analysis provides details on the CV safety of erenumab across different cardiovascular risk profiles.
Mascarella et al. [12]	2024	Multicenter, prospective, real-world, observational study	155 patients	64 ± 3.1 years (range: 60-80)	Both episodic (47 patients, 31.0%) and chronic (105 patients, 69.0%) migraine	Erenumab, galcanezumab, or fremanezumab	Erenumab: 40 patients; galcanezumab: 47 patients; fremanezumab: 68 patients	Erenumab: 70 mg (option to titrate to 140 mg); galcanezumab: 240 mg loading dose, then 120 mg monthly; fremanezumab: 225 mg monthly or 675 mg quarterly	Subcutaneous injection	12 months	Blood pressure changes (systolic and diastolic); newly or worsened hypertensive patients (NWHP); cardiovascular safety	No significant systolic or diastolic BP changes at any time point (all p > 0.05). 20/155 (12.9%) patients were NWHP; 11/20 had prior hypertension. A higher baseline BP value is associated with increased BP (p = 0.002).	Treatment with anti-CGRP mAbs over one year does not significantly affect BP in patients aged ≥60, nor does it increase hypertension incidence compared to general population trends. Continuous monitoring is recommended.
Wang et al. [13]	2023	Retrospective cohort study	69,589 patients	Non-hypertensive: anti-CGRP: 43.4 (10.5) years, topiramate: 40.7 (10.6) years; hypertensive: anti-CGRP: 54.4 (11.2) years, topiramate: 53.6 (11.8) years	Both episodic and chronic migraine (53.2% of the anti-CGRP group had chronic migraine vs. 16.6% of the topiramate group)	Anti-CGRP treatment (erenumab, fremanezumab, galcanezumab, rimegepant, atogepant) vs. topiramate	Anti-CGRP: 18,880 patients (27.1%); topiramate: 50,709 patients (72.9%)	Not specified for individual agents	Subcutaneous injection for mAbs, oral for small molecules	Median follow-up: 0.9 years (IQR: 0.3-2.0)	Blood pressure trajectory, hypertension incidence, and number of antihypertensive medications	Non-hypertensive patients: No significant difference in BP changes between groups (slope 0.48 vs. 0.39, p = 0.21); Hypertensive patients: the anti-CGRP group had a 3.7% annual increase in antihypertensive medications (RR = 1.037, 95% CI: 1.025-1.048).	Anti-CGRP treatment is safe regarding blood pressure in patients without hypertension. For those with baseline hypertension, anti-CGRP treatment resulted in a small but persistent increase in antihypertensive medications, indicating exacerbation of hypertension.

Quality Assessment

The quality assessment of included studies using the NOS revealed generally moderate to high methodological quality across the five studies (Table 2). The pooled clinical trial analyses (Dodick et al., 2021; Kudrow et al., 2023) demonstrated high quality with robust randomization, adequate blinding, and comprehensive outcome reporting [10,11]. The prospective studies (de Vries Lentsch et al., 2022; Mascarella et al., 2024) showed moderate quality with adequate participant selection and outcome assessment but limited comparability controls [9,12]. The large retrospective cohort study (Wang et al., 2023) achieved high quality through adequate sample size, appropriate exposure definition, and comprehensive follow-up, though retrospective design limitations were noted [13].

**Table 2:** Quality assessment summary table. NOS: Newcastle-Ottawa Scale; RCT: randomized controlled trial; BP: blood pressure.

Study	Year	Study design	NOS score	Quality rating	Selection	Comparability	Outcome	Key strengths	Limitations
de Vries Lentsch et al. [9]	2021	Pooled RCT analysis	8/9	High	4/4	2/2	2/3	Large sample, randomized design, standardized outcomes	Short follow-up duration
Dodick et al. [10]	2023	Post-hoc pooled analysis	8/9	High	4/4	2/2	2/3	Extended follow-up, cardiovascular focus	Post-hoc design, selected populations
Kudrow et al. [11]	2022	Prospective follow-up	6/9	Moderate	3/4	1/2	2/3	Prospective design, direct BP measurements	Small sample, no control group
Mascarella et al. [12]	2024	Prospective observational	6/9	Moderate	3/4	1/2	2/3	Real-world setting, elderly population	Small sample, single-arm design
Wang et al. [13]	2023	Retrospective cohort	7/9	High	3/4	2/2	2/3	Large sample, comparative design, long-term follow-up	Retrospective design, administrative data

Discussion

This systematic review provides a comprehensive evaluation of hypertension occurrence in patients receiving CGRP mAbs for migraine prevention, revealing a complex and evolving safety profile that warrants clinical attention. The synthesis of evidence from five diverse studies demonstrates that while the overall risk of hypertension appears modest, subtle but clinically meaningful effects on blood pressure regulation may occur in certain patient populations. The findings suggest a differential risk profile between CGRP receptor antagonists and ligand-targeting antibodies. de Vries Lentsch et al.'s study demonstrated that erenumab, which targets the CGRP receptor, produced greater blood pressure increases compared to fremanezumab, a ligand-targeting antibody [9]. This observation aligns with theoretical considerations regarding CGRP's physiological role in cardiovascular homeostasis. The CGRP receptor is more directly involved in vascular smooth muscle relaxation and compensatory vasodilation during hypertensive states, potentially explaining why receptor blockade may have more pronounced cardiovascular effects than ligand neutralization [7].

Wang et al.'s retrospective cohort study provides particularly valuable insights into real-world clinical practice, demonstrating that patients with baseline hypertension experienced a 3.7% annual increase in antihypertensive medication requirements when treated with anti-CGRP therapies [13]. This finding suggests that CGRP inhibition may exacerbate existing hypertension rather than causing de novo hypertension in normotensive individuals. The absence of significant blood pressure changes in non-hypertensive patients supports this hypothesis and provides reassurance for the majority of migraine patients who do not have pre-existing cardiovascular comorbidities. The temporal aspects of hypertension development also merit consideration. de Vries Lentsch et al.'s study showed progressive blood pressure increases over the treatment period, with 3.7% of patients with normal baseline blood pressure requiring antihypertensive treatment after erenumab initiation [9]. This pattern suggests that hypertensive effects may be cumulative rather than immediate, emphasizing the importance of long-term monitoring strategies in clinical practice.

Age-related considerations emerge from Mascarella et al.'s study, which focused on patients aged 60 and older [12]. Despite this population's inherently higher cardiovascular risk, the study found no significant blood pressure changes, though 12.9% of patients were classified as having newly or worsened hypertensive patterns. This finding suggests that age alone may not be a primary risk factor for CGRP-associated hypertension, though the relatively small sample size limits definitive conclusions. The clinical trial data from Dodick et al. and Kudrow et al. provide important context by demonstrating low incidence rates of hypertension adverse events in controlled settings [10,11]. However, the short duration of most clinical trials (12-24 weeks) may not capture the full spectrum of cardiovascular effects that become apparent during extended real-world use. The post-marketing surveillance data showing 362 hypertension adverse events in over 245,000 patient-years of exposure (0.144 per 100 patient-years) provides a more comprehensive long-term perspective [10].

The heterogeneity in outcome definitions across studies highlights the need for standardized approaches to cardiovascular safety monitoring. While some studies focused on blood pressure measurements, others emphasized adverse event reporting or medication initiation, making direct comparisons challenging. This methodological variability underscores the importance of developing consensus guidelines for cardiovascular monitoring in patients receiving CGRP-targeting therapies. From a mechanistic perspective, these findings align with CGRP's established role in cardiovascular physiology. CGRP functions as a potent vasodilator and plays a compensatory role during hypertensive states by promoting vascular relaxation and increasing blood flow [14]. Inhibiting this pathway may impair these protective mechanisms, particularly in patients with pre-existing cardiovascular compromise or those with genetic predispositions to hypertension.

Limitations and future directions

This systematic review has several important limitations that should be considered when interpreting the findings. The heterogeneity in study designs, outcome definitions, and follow-up durations precluded quantitative meta-analysis, limiting the ability to derive precise risk estimates. The included studies employed varying definitions of hypertension, ranging from blood pressure measurements to adverse event reporting, making direct comparisons challenging. Sample sizes varied considerably, with some studies potentially underpowered to detect meaningful differences in hypertension incidence. The relatively short follow-up periods in most clinical trials may not capture the full spectrum of cardiovascular effects that develop during extended treatment. Additionally, the exclusion of patients with significant cardiovascular comorbidities from many clinical trials may limit the generalizability of findings to real-world populations with higher baseline cardiovascular risk.

Future research should prioritize standardized cardiovascular monitoring protocols across CGRP mAb studies. Long-term prospective studies with extended follow-up periods are needed to better characterize the temporal relationship between CGRP inhibition and hypertension development. Comparative effectiveness studies directly comparing different CGRP-targeting agents would help clarify differential cardiovascular risks. Additionally, mechanistic studies investigating the relationship between CGRP pathway inhibition and blood pressure regulation could inform risk stratification strategies. Development of clinical guidelines for cardiovascular monitoring in patients receiving CGRP therapies represents an important clinical priority.

## Conclusions

This systematic review demonstrates that CGRP mAbs present a generally favorable cardiovascular safety profile, though clinically relevant hypertensive effects may occur in specific patient populations. The evidence suggests differential risk profiles between receptor antagonists and ligand-targeting antibodies, with erenumab showing a greater propensity for blood pressure elevation. Patients with pre-existing hypertension appear at higher risk for exacerbation of their condition, while normotensive individuals demonstrate minimal cardiovascular impact. The temporal pattern of hypertension development appears cumulative rather than immediate, emphasizing the importance of long-term monitoring strategies. Healthcare providers should implement regular blood pressure monitoring for patients receiving CGRP therapies, particularly those with baseline cardiovascular risk factors or pre-existing hypertension. While these findings should not preclude the use of CGRP mAbs in appropriate patients, they underscore the need for individualized risk-benefit assessments and proactive cardiovascular monitoring in clinical practice. Future research should focus on standardized monitoring protocols and long-term comparative effectiveness studies to further refine our understanding of cardiovascular safety across different CGRP-targeting agents.
